# Changes in Residential Greenspace and Birth Outcomes among Siblings: Differences by Maternal Race

**DOI:** 10.3390/ijerph20186790

**Published:** 2023-09-21

**Authors:** Samantha Gailey

**Affiliations:** 1 Department of Forestry, Michigan State University, East Lansing, MI 48824, USA; sgailey@msu.edu; 2 Department of Public Health, Michigan State University, Flint, MI 48502, USA

**Keywords:** greenspace, pregnancy, health disparities, perinatal health, birth outcomes, natural environment, nature

## Abstract

Growing research investigates the perinatal health benefits of greenspace in a mother’s prenatal environment. However, evidence of associations between residential greenspace and birth outcomes remains mixed, limiting the relevance this work holds for urban policy and greening interventions. Past research relies predominantly on cross-sectional designs that are vulnerable to residential selection bias, and rarely tests effect modification by maternal race/ethnicity, which may contribute to heterogeneous findings. This study uses a rigorous, longitudinal sibling comparison design and maternal fixed effect analyses to test whether increases in maternal exposure to residential greenspace between pregnancies precede improved birth outcomes among non-Hispanic (NH) white (*n* = 247,285) and Black (*n* = 54,995) mothers (mean age = 28 years) who had at least two consecutive live births in California between 2005 and 2015. Results show that increases in residential greenspace correspond with higher birthweight (coef. = 75.49, 95% CI: 23.48, 127.50) among Black, but not white (coef. = −0.51, 95% CI: −22.90, 21.90), infants. Additional analyses suggest that prior evidence of perinatal benefits associated with residential greenspace among white mothers may arise from residential selection; no such bias is observed for Black mothers. Taken together, these findings support urban greening initiatives in historically under-resourced neighborhoods. Efforts to evenly distribute residential greenspace may reduce persistent racial disparities in birth outcomes, an important step towards promoting health equity across the life course.

## 1. Introduction

In the US, the incidence of adverse birth outcomes including preterm birth (PTB) and low birthweight (LBW) exceed those of all other high-income countries. PTB and LBW—defined as delivery at less than 37 weeks of gestational age and birthweight of less than 2500 g—increase infant mortality risk, impair childhood development, reduce earnings and educational attainment into adulthood, and impose substantial hospital-based obstetric costs [1,2,3]. Given the deleterious life course sequelae and financial burdens associated with PTB and LBW, these adverse birth outcomes command considerable attention from scholars and policymakers. Despite mounting clinical efforts, PTB and LBW rates remain high, particularly among racial/ethnic minorities. Compared to non-Hispanic (NH) white birthing people, Black birthing people are more than twice as likely to deliver preterm and low-weight births, portending a disadvantage for racially minoritized infants from the first moments of life [4].

Research on PTB, LBW, and other perinatal health outcomes (e.g., small-for-gestational age; SGA) indicates complex etiologies that involve interactions between individual biological and behavioral factors and environmental conditions [1,2,5]. Growing evidence suggests that neighborhood context lays the foundation for lifelong health and development as early as conception [6]. Over the last decade, researchers have explored the benefits of increasing residential greenspace as a potential intervention to improve birth outcomes and reduce disparities that persist across the life course [7,8]. This possibility has gained interest in the scholarly community given that greenspace shows well-documented benefits for widespread physical health outcomes [9,10,11], appears more feasible and less expensive to modify relative to other environmental amenities, and remains underrepresented in low-income and minoritized areas [12].

However, evidence on associations between residential greenspace and birth outcomes appears mixed. Inconsistent findings limit the relevance this work holds for policies and interventions that aim to improve urban greenspace and, ultimately, promote perinatal health in under-resourced neighborhoods. Researchers have proposed plausible explanations for equivocal results, including that: (1) maternal characteristics associated with both place of residence and perinatal health may explain positive associations between greenspace and birth outcomes [13,14] and (2) maternal sociodemographic characteristics may modify the effects of greenspace on birth outcomes [15,16]. Differentiating these plausible causes of heterogeneity is necessary to understand the effects of greenspace on perinatal health and advance urban greening efforts. Thus, this study aimed to use rigorous longitudinal methods to minimize residential self-selection bias and assess whether associations between changes in greenspace and birth outcomes differ by maternal race/ethnicity.

### 1.1. Neighborhood Greenspace and Birth Outcomes

Neighborhood greenspace may improve birth outcomes through multiple psychophysiological, behavioral, and environmental pathways, categorized into domains of restoration, instoration, and mitigation [11]. Restorative environments theories, including Stress Reduction Theory and Attention Restoration Theory, posit that, under antecedent conditions of stress and mental fatigue, greenspace exposure improves mood, enhances attention, and reduces perceived and physiological stress [17,18]. Accumulating experimental evidence over the past three decades supports these hypotheses [19]. Observational studies that assess stress biomarkers (e.g., cortisol) as a function of longer-term exposure to residential environments also support the notion that surrounding greenspace serves a long-term restorative function [20].

Prenatal maternal stress may increase the risk of adverse birth outcomes [21]. A broad range of individual and ecological stressors appear to affect intrauterine growth and the timing of parturition [22,23,24]. Findings from diverse studies indicate that stress triggers a physiological response along the maternal hypothalamic–pituitary–adrenal (HPA) axis, which may result in elevated intrauterine and fetal cortisol concentrations and/or perturb normal placental function [25]. Neighborhood greenspace, as a restorative setting, may thus mitigate the extent to which exposure to stressors adversely affects the course of pregnancy.

Natural environments may also confer ecological benefits, or ‘ecosystem services’, that improve perinatal health by mitigating adverse environmental exposures [9,11]. Much work on this topic has focused on the relation between residential greenspace and air pollution exposure among pregnant populations [26,27]. Studies have found stronger inverse associations between greenspace and PTB among mothers who had greater exposure to air pollution during pregnancy [28] and shown that reductions in fine particulate matter mediate the association between greenspace and LBW [29]. Beyond restoration and mitigation, residential greenspace may also serve an ‘instorative’ function by encouraging behaviors like physical activity and social interactions that promote health more broadly [11].

Building on evidence of these health-promoting mechanisms, growing epidemiologic research examines whether greenspace in a mother’s prenatal environment correlates with more favorable birth outcomes [7,30,31]. Although this work provides some evidence of perinatal health benefits [32,33], findings on outcomes associated with residential greenspace do not converge. One review of this literature, for example, reports evidence of positive associations between greenspace and infant birthweight but null relations with gestational age [30]. Other studies have found protective associations between greenspace and preterm and growth-restricted births, but not birthweight [34], or null relations across all birth outcomes [13].

### 1.2. Residential Self-Selection Bias

Biases in past work may contribute to inconsistent findings on greenspace–birth outcome associations. Most studies examining perinatal benefits associated with neighborhood greenspace use observational and cross-sectional designs, which appear vulnerable to residential self-selection bias [30]. The selective movement of mothers into greener neighborhoods on the basis of preexisting health and correlated social factors (e.g., income, education, race/ethnicity) presents a rival explanation for previously observed protective associations between neighborhood greenspace and birth outcomes [14,35]. The spatial concentration of healthier mothers in neighborhoods with more greenspace may reflect residential selection, rather than a causal effect of greenspace on health [36,37]. For example, Gailey [14] recently found that mothers with lower pre-pregnancy BMI and higher infant birthweights (proxies for better health) moved to greener neighborhoods over time. This form of selection appears less pronounced among Black mothers, who face considerable housing choice constraints imposed by discrimination and other forces of structural racism [14].

Recent work by Margerison et al. examined residential self-selection bias by testing whether maternal characteristics associated with both residential greenspace exposure and birth outcomes could explain protective associations. The results of adjusted cross-sectional analyses indicated that residential greenspace corresponded with a lower risk of PTB and increased birthweight. However, the results of longitudinal within-mother analysis (i.e., maternal fixed effects), which further controlled for unobserved maternal characteristics, no longer rejected the null. Taken together, these findings suggest that differential selection into greener neighborhoods among mothers with lower risks of adverse birth outcomes may explain past findings on greenspace and perinatal health [13].

### 1.3. Effect Modification by Maternal Race/Ethnicity

Alternate explanations for inconsistent findings include that individual factors may modify associations between residential greenspace and birth outcomes. Race/ethnicity, income, and education (among other characteristics) may influence a mother’s ability to access and use green spaces in the residential environment to improve health. The racial, ethnic, and socioeconomic composition of a study sample may thus influence the direction or magnitude of associations between greenspace and perinatal health, producing heterogeneous results [15]. Supporting this notion, several studies have shown that income and education levels modify the association between residential greenspace and birth outcomes, such that less-educated and lower-income mothers exhibit stronger protective associations [16,26,38,39,40].

Some work suggests that maternal race/ethnicity may also modify associations between neighborhood greenspace and perinatal health. Although empirical evidence remains mixed, research documents plausible pathways through which greenspace may differentially improve birth outcomes among racially minoritized mothers [16,30,40,41,42]. For example, studies consistently find higher levels of maternal stress [43] and exposure to air pollution [44,45]—two key risk factors for adverse birth outcomes—among NH Black mothers [46,47]. Evidence that greenspace mitigates these factors [19,28,29,48] suggests that residential greenspace may confer greater perinatal health benefits to Black, compared to white, mothers. 

At least one prior study found that race/ethnicity modifies the association between residential greenspace and birth outcomes. Using cross-sectional data in the UK, Dadvand et al. [16] showed that higher levels of greenspace in the prenatal environment vary with higher infant birthweight among mothers of White British, but not Pakistani, origins. However, the extent to which these findings generalize to births among white and Black mothers remains unclear [30]. Studies in the US [40] and Canada [41] found no evidence of differences in associations between greenspace and birth outcomes by race or ethnicity.

### 1.4. Current Study and Hypotheses

This study examines racial/ethnic differences in longitudinal associations between residential greenspace and birth outcomes, including infant birthweight and SGA, among siblings born in California between 2005 and 2015. I use several analytic strategies with increasing levels of rigor to investigate potential residential selection bias [13,14] and stratify analyses by maternal race/ethnicity to examine effect modification [16]. Building on recent longitudinal studies [13,49], I focus on mothers who remain in the same neighborhood across births, given that within-neighborhood changes in greenspace may replicate an intervention. Moreover, given that Black mothers in the US exhibit the highest risks of adverse birth outcomes and may benefit to a greater extent from increased greenspace exposure than other racial/ethnic subgroups, this study focuses its hypotheses and analyses on NH Black mothers.

I hypothesized that, in cross-sectional analyses adjusted for observed maternal and neighborhood characteristics, residential greenspace would correspond with higher birthweight and lower odds of SGA among all racial/ethnic subgroups. However, I predicted that sibling comparison (i.e., maternal fixed effects) analyses, which further control for unobserved time-invariant characteristics of mothers (including residential selection factors), would show protective effects of greenspace on births only among NH Black mothers. Moreover, I expected the results of maternal fixed effects analyses to hold for mothers who did not move between births, such that within-neighborhood increases in residential greenspace would precede higher birthweight and lower odds of SGA among NH Black mothers.

## 2. Materials and Methods

### 2.1. Data and Sample

I retrieved data on all live births in California (CA) between January 2005 and December 2015 from the California Department of Public Health (CDPH) Birth Cohort Files (BCFs). The CA BCF contains data recorded from the US Standard Certificate of Birth, including maternal and infant health and demographic characteristics, for more than 99.99% of births in California. The CA BCF also includes information on mothers’ residential addresses at the time of birth, which I geocoded and linked to census tract-level measures of greenspace and disadvantage (described below). CDPH records the race (a 7-category variable, including American Indian or Alaskan Native [AIAN]; Asian; Black or African American; Native Hawaiian or Other Pacific Islander [NHOPI]; white; more than one race; and unknown or not stated) and ethnicity (a 3-category variable, including Hispanic or Latino; not Hispanic or Latino; and origin unknown or not stated) of the mother, which I used to classify into the following categories, consistent with the literature: non-Hispanic (NH) white, Black, Hispanic, Asian, and ‘other’ (including small percentages of AIAN, NHOPI, more than one race reported, and unknown or not stated race or ethnicity). The State of California and the University of California, Irvine, approved the study (IRB protocol approval # 13-06-1251 and 2013-9716, respectively).

### 2.2. Sibling Linkage Strategy

The CA BCF arrays data at the infant level and does not include unique maternal identifiers. To identify live births to the same mother, I used Link Plus (version 3.0), an open-source probabilistic record linkage program developed by the Division of Cancer Prevention within the Centers for Disease Control and Prevention (CDC). The CA BCFs for the years 2005 to 2015 include records for 5,814,502 live births. I excluded records of non-singleton birth events, given that multiple births interfere with the sibling linkage process (*n* = 185,930). I also excluded birth records missing data on the mother’s date of birth (*n* = 2183), last name (*n* = 24,502), and first name (*n* = 2934) as I required this information to match siblings delivered by the same mother. To develop the sibling linkage algorithm (described in more detail in the Supplementary Material), I used Link Plus to pair mothers with the same date of birth and assigned a match score to potential sibling pairs based on the similarity of the mother’s first and last names and father’s date of birth. I performed a manual review of the potential pairs to ensure that the date of birth in the record of sibling 1 corresponded with the date of last delivery in the record of sibling 2. The resulting dataset included 1,340,676 sibling pairs (i.e., mothers with at least two consecutive live births in California) from 2005 to 2015.

### 2.3. Geocoding

The CA BCF includes data on mothers’ addresses, zip codes, and city of residence. I geocoded maternal residential addresses at the time of each birth (i.e., for each sibling) to derive latitude and longitude point coordinates using ArcGIS software version 10.4 (Redlands, California). I located addresses using a 2013 street directory and joined point coordinates with census tracts, a proxy for neighborhoods, based on 2010 US Census geography. I excluded sibling pairs if the maternal residential address provided in one or both sibling birth records did not reach a minimum location match score of 80%; with unknown, missing, or non-California census tracts; or if mothers lived in non-urban tracts (*n* = 97,276). Urban areas, according to the Census Bureau, encompass densely settled neighborhoods comprising at least 50,000 residents. Based on this definition, many neighborhoods typically identified as suburbs are also categorized as urban (US Census Bureau, 2010). This distinction is substantively relevant, as relations between greenspace and health (including birth outcomes) appear to differ by urbanicity, with more protective associations generally observed in urban areas. Elevated exposure to psychosocial and environmental stressors—risk factors that greenspace may mitigate—could explain the stronger effects of greenspace among urban populations [50].

The exclusion process yielded an analytic sample of 922,263 mothers with at least two consecutive live births, and who lived in urban census tracts in California, from 2005 to 2015. Subgroups for race/ethnicity-specific analyses included 247,285 NH white mothers, 54,995 Black mothers, 463,707 Hispanic mothers, and 136,707 Asian mothers. I did not include ‘other’ race/ethnicity in subgroup analyses, given that this category represents a highly heterogeneous population.

### 2.4. Measures

#### 2.4.1. Neighborhood Characteristics

Neighborhood Greenspace. I retrieved neighborhood greenspace data from the NOAA Climate Data Record (CDR) Normalized Difference Vegetation Index (NDVI) remote sensing product [51]. The NDVI CDR measures and summarizes surface vegetation activity across the globe and is widely used in epidemiological studies [41,49,52]. NDVI is calculated using the spectral bands in the red and near-infrared wavelengths derived from Advanced Very High Resolution Radiometer (AVHRR) data obtained from 8 NOAA polar-orbiting satellites. The NDVI CDR product generates daily NDVI values with a spatial resolution of 30 m (a 0.05° × 0.05° grid). I used Google Earth Engine to create an average NDVI measure at the census tract–year level (i.e., one measure per census tract per year) in California between 2005 and 2015. NDVI values range from −1 to 1, where higher values reflect a greater density of greenness. Negative values correspond to blue spaces (i.e., water bodies), which may mathematically average out green spaces, despite potentially exerting similar effects [11,53]. Consistent with recent studies of greenspace and physical health, I re-coded negative NDVI values to zero [28,54]. I linked census tract–year-level NDVI to infant births by maternal residential census tract and the calendar year at the time of birth. For example, for births on 1 May 2013, I assigned the corresponding mean NDVI value for the year 2013 in the mother’s census tract of residence.

I elected to include satellite images captured during the full calendar year to generate mean NDVI values, given that California (which is characterized by a Mediterranean-like climate) exhibits relatively little within-region variation in vegetation across different seasons. By contrast, as the third largest US state by area, California shows considerable between-region variation in vegetation [55]. The central and southwest regions, for example, comprise large desert areas with dry climates throughout the year. Since NDVI captures the density of green vegetation, these regions have low NDVI values relative to California’s coastal and northern regions. Notably, greenspace in desert-like regions may influence residents’ health comparably to greenspace in non-desert settings [56], supporting the validity of NDVI as a greenspace measure across California’s diverse regions. Though beyond the scope of this study, ‘brown’ spaces (characteristic of most nature in desert-like settings) also appear to promote health through similar pathways as greenspace [57].

Neighborhood Disadvantage. I merged individual-level data and measures of neighborhood greenspace with a census tract-level measure of neighborhood disadvantage. Consistent with past work, I calculated an index of neighborhood disadvantage using six variables retrieved from the 2010 U.S. Decennial Census: the proportion of households with income < USD 15,000, the proportion of households with income ≥ USD 50,000 (reverse coded), the proportion of families in poverty, the proportion of households receiving public assistance, the total unemployment rate, and the proportion of vacant housing units (Cronbach’s alpha = 0.92) [58]. I standardized each variable and calculated the mean of the standardized variables to construct the census tract-level index, expressed as a continuous variable. Values can be interpreted as the number of standard deviations above or below the California urban census tract-level mean.

#### 2.4.2. Individual Characteristics

Sociodemographic Characteristics. The CA BCFs for the years 2005 to 2015 include information on the demographic and socioeconomic characteristics of the mother. In addition to maternal race and ethnicity (described above), CDPH collects data on maternal age (categorized as <20, 20–24, 25–29, 30–34, 35–39, and 40 years or older), previous live births (i.e., parity, categorized as nulliparous [0 previous live births], primiparous [1 previous live birth], and multiparous [2 or more previous live births]), highest educational attainment (categorized as less than high school, high school graduate or GED, some college, and unknown or not stated), and insurance provider (categorized as public, private, and other [including self-pay, Indian Health Service, other governmental source, and unknown or not stated]). This set of time-varying sociodemographic characteristics represent potential confounders as they may influence both a mother’s residential exposure to greenspace and her birth outcomes. As such, these measures were included as covariates in adjusted analyses (Models 2–6, described in ‘Analytic Approach,’ below).

Birth Outcomes. Outcomes analyzed in the main text of this study include (1) birthweight in grams (g), a continuous variable, and (2) small-for-gestational age (SGA), a binary variable. I retrieved data on birthweight and gestational age at birth based on the last menstrual period from birth certificates. SGA gauges infant growth restriction while adjusting for the timing of parturition. To derive SGA, I used sex- and gestational-week-specific US national reference charts for birthweight to classify each infant birth into corresponding birthweight percentiles [59]. I then defined SGA infants as those with birthweight for a gestational age less than the 10th percentile. Continuous birthweight (g) serves as the primary outcome, as the statistical modeling of binary outcomes using a maternal fixed effects approach includes only siblings who are doubly discordant with respect to the exposure and outcome of interest, reducing statistical power with which to detect the effects of greenspace on birth outcomes [60,61]. However, I also chose to examine SGA, as it takes into account gestational length, which may have a distinct etiology from birthweight [59]. In addition, to increase comparability with past studies on greenspace and birth outcomes, I examined preterm birth (PTB; delivery at less than 37 weeks gestational age) and low birthweight (LBW; birthweight less than 2500 g), the results of which are available in the Supplementary Material.

Movers and Stayers. I defined movers as mothers whose census tract changed between their first and second birth (*n* = 433,079); stayers remained in the same census tract across births (*n* = 489,184), although they could have moved within the tract. The types of changes in greenspace that movers and stayers experience generally differs: movers experience between-neighborhood changes in greenspace (induced by moving to a new neighborhood) while stayers experience within-neighborhood changes in greenspace (induced by a variety of factors, including natural variation in vegetation over time and person-made changes related to the creation, enhancement, destruction, or deterioration of parks, green parkways, street trees, and other forms of greenery).

#### 2.4.3. Analytic Approach

I estimated associations between residential greenspace and birth outcomes, overall (i.e., including mothers of all racial/ethnic identities) and separately for NH white, Black, Hispanic, and Asian mothers using two primary approaches. I initially performed cross-sectional analyses to examine relations between residential greenspace and birth outcomes (birthweight, SGA, PTB, and LBW). Next, I leveraged longitudinal data on a mother’s first and second births and used a ‘within-mother’ (i.e., maternal fixed effects) approach to estimate the effects of changes in greenspace on birth outcomes.

In contrast to cross-sectional analyses, maternal fixed effects models include a mother-specific indicator variable to control for time-invariant characteristics of the mother [61]. These analyses estimate the influence of a change in residential greenspace on birth outcomes using a within-mother counterfactual—that is, the birth outcome of a sibling born under different conditions of greenspace in the residential environment. By comparing births within the same mother, this approach controls for unobserved confounders that remain stable across births, including factors that may influence residential selection.

For all models, I first estimated associations between neighborhood greenspace and birth outcomes in the full analytic sample of mothers (i.e., across racial/ethnic groups) with at least two births in California between 2005 and 2015. I then stratified analyses by race/ethnicity to assess greenspace–birth outcome relations separately for NH white, Black, Hispanic, and Asian mothers. I used linear regression models to estimate associations between NDVI (modeled continuously) and birthweight (g) and logistic regression models to estimate associations between NDVI (modeled as quartiles) and the odds of SGA, PTB, and LBW. All statistical analyses were performed in SAS version 9.4 (Cary, NC, USA).

#### 2.4.4. Cross-Sectional Analyses

Model 1 assessed the unadjusted association between residential greenspace and birth outcomes at time 2 (i.e., sibling 2). Model 2 controlled for individual- and neighborhood-level covariates that could affect both residential greenspace and birth outcomes, including maternal age, education, parity, insurance status, and neighborhood disadvantage, as well as the year of birth (to control for secular trends). Model 3 used a pooled cross-sectional approach to assess relations between residential greenspace and birth outcomes among siblings born at time 1 (i.e., sibling 1) and time 2 (i.e., sibling 2) in the within-mother sample. Given that these analyses include multiple births to the same mother, I clustered standard errors by mother and, building on Model 2, adjusted for year of birth and individual- and neighborhood-level covariates. However, unlike the longitudinal within-mother analyses described below, Model 3 does not control for unobserved time-invariant maternal characteristics.

#### 2.4.5. Within-Mother Analyses

All within-mother analyses included a mother-specific indicator variable to control for time-invariant maternal characteristics and included birth data for both siblings. Model 4 examined the within-mother association between changes in residential greenspace and birth outcomes among siblings, adjusted for time-varying individual and neighborhood characteristics (maternal age, education, parity, insurance status, neighborhood disadvantage, and year of birth). Models 5 and 6 restricted within-mother analyses to mothers who remained in the same neighborhood (stayers) and mothers who moved to a different neighborhood (movers) between births, respectively.

## 3. Results

Table 1 shows descriptive statistics for mothers and singleton live births at time 2 (i.e., sibling 2) in California between 2005 and 2015. The full analytic ‘within-mother’ sample (left column) displays the characteristics of all mothers with at least two consecutive births over the study period (*n* = 922,263); the ‘stayers only’ sample (right column) shows the characteristics of mothers within the full sample who remained in the same neighborhood across births. Both the full within-mother sample and the restricted stayers only sample include mothers from racially and socioeconomically diverse backgrounds. Most mothers in the full sample (Table 1A, left column) identified as Hispanic (50.28%) or NH white (26.81%), attained at least some college education (52.18%), and had private health insurance (49.88%) at the time of their second birth. A higher proportion of mothers who stayed in the same neighborhood across births reported at least some college education and had private health insurance (Table 1A, right column). This result converges with evidence that higher socioeconomic status groups tend to move less frequently.

Table 1 also shows the characteristics of NH white mothers (*n* = 247,285) (Table 1B) and NH Black mothers (*n* = 54,995) (Table 1C) and their births at time 2. Compared to NH white mothers, a higher proportion of NH Black mothers reported having public health insurance and attained less than a high school education. Infants born to NH Black mothers were substantially lighter and spent less time in gestation on average than infants born to NH white mothers. In addition, NH Black mothers lived in neighborhoods with higher levels of disadvantage and lower levels of greenspace.

Table 2 shows the distribution of mothers by exposure to neighborhood greenspace, categorized into quartiles, at time 1 and time 2 in the full analytic sample (Table 2A) and separately for NH white (Table 2B) and NH Black (Table 2C) mothers. At the time of both births, more NH white mothers lived in neighborhoods with high levels of greenspace, and fewer lived in neighborhoods with low levels of greenspace, than NH Black mothers. For example, approximately 17% of NH white mothers lived in neighborhoods with very low (quartile [Q] 1) greenspace at time 1 compared to more than 38% of NH Black mothers. Across racial/ethnic subgroups, a slightly higher proportion of mothers lived in neighborhoods with high (Q3) and very high (Q4) levels of greenspace at time 2 compared to time 1, indicating that more mothers moved to greener, rather than less green, neighborhoods between births.

Table S1 (in the Supplementary Material) shows mean changes in neighborhood greenspace between births in the within-mother and stayer samples by race/ethnicity. NH Black mothers, on average, moved to slightly greener neighborhoods between births (mean change = 0.03). NH white mothers, conversely, moved to slightly less green neighborhoods (mean change = −0.01). Large standard deviations in both the within-mother and stayer samples indicate substantial variation in the change in greenspace that mothers experienced between births.

### 3.1. Birthweight Analyses

#### 3.1.1. Cross-Sectional Results

Table 3A shows the results of cross-sectional analyses (Models 1–3) predicting birthweight as a function of residential greenspace in the full analytic sample and separately for NH white, Black, Hispanic, and Asian mothers. Across cross-sectional models, I found positive associations between residential greenspace and birthweight among all mothers except those of Asian identity. I observed the strongest cross-sectional associations among NH Black and Hispanic mothers. For example, Model 1 (not adjusted for covariates) indicates that a one-unit increase in NDVI corresponds with a 120.33 g increase in birthweight among births to NH Black mothers (95% CI: 88.63, 152.03) and a 110.18 g increase in birthweight among births to Hispanic mothers (95% CI: 98.79, 121.57), relative to a 59.09 g increase in birthweight among NH white births (95% CI: 42.81, 75.38). Adjusting for individual- and neighborhood-level covariates (Model 2) attenuates point estimates but inference does not change. Model 3, which estimates cross-sectional relations between residential greenspace and birth outcomes pooled across both siblings, also shows positive greenspace–birthweight associations among NH Black (coef. = 92.60, 95% CI: 67.68, 117.51), Hispanic (coef. = 93.72, 95% CI: 84.63, 102.81), and NH white (coef. = 68.08, 95% CI: 55.61, 81.56) mothers.

#### 3.1.2. Within-Mother Results

Consistent with my hypothesis, maternal fixed effects analyses (Table 3B) comparing birth outcomes within the same mother (i.e., with different levels of exposure to greenspace across births) indicate that the positive greenspace–birthweight result holds only for NH Black mothers (coef. = 74.59, 95% CI: 23.48, 127.50) (Model 4). Notably, even Hispanic mothers, who showed cross-sectional associations similar in magnitude to NH Black mothers, no longer show statistically detectable associations. The results of the within-mother analyses in mothers who remain in the same neighborhood (stayers, Model 5) and who move between births (movers, Model 6) further show that increases in greenspace correspond with increases in birthweight only among NH Black mothers.

**Figure 1:**
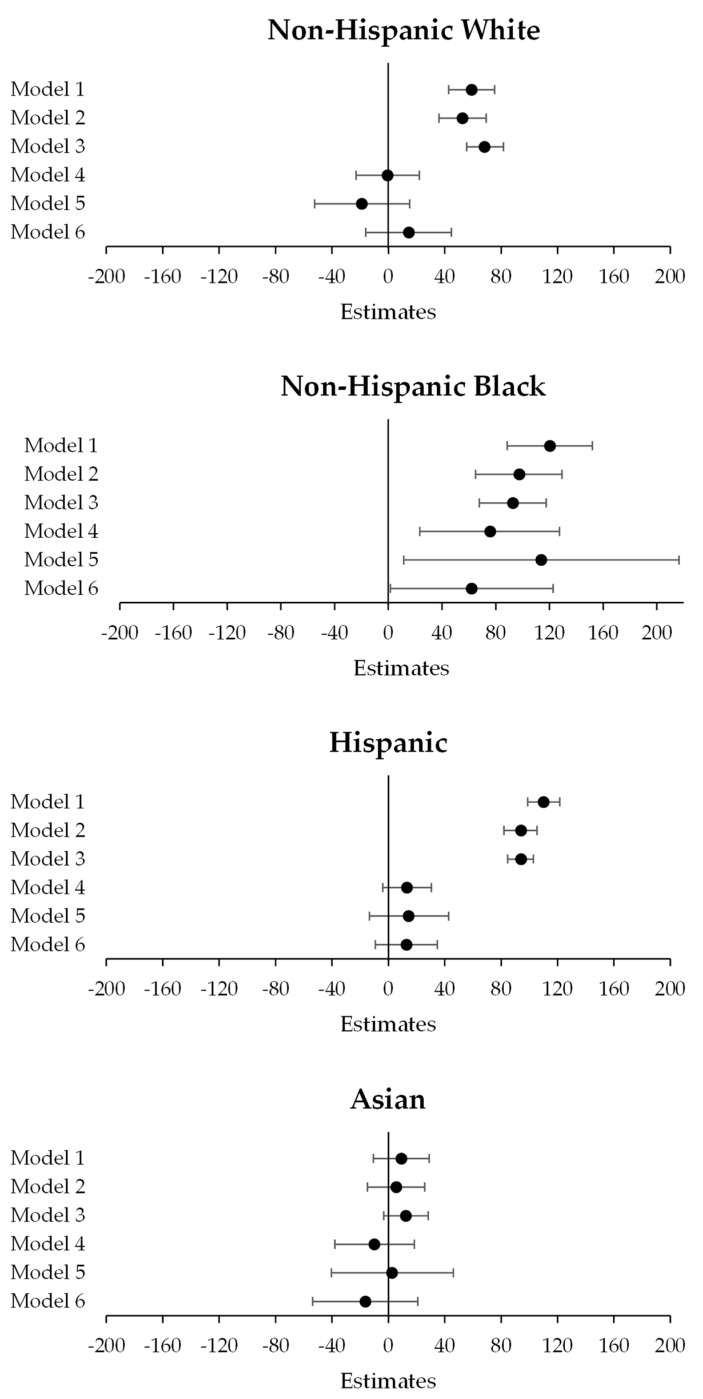
Forest plot summarizing results of linear regression analyses (Models 1–6) by race/ethnicity.

Figure 1 displays the results of Models 1–6 (among NH white, Black, Hispanic, and Asian mothers) in forest plots. Supplementary Tables S2 and S3 additionally show associations between birthweight and a 0.1-unit increase in NDVI (Table S2) and an interquartile range (IQR) increase in NDVI (Table S3), rather than a full one-unit increase in NDVI (which represents an unlikely change from 0% to 100% neighborhood greenspace).

### 3.2. SGA Analyses

Models estimating associations between neighborhood greenspace (in quartiles) and SGA show a different pattern of results (Table 4) than those estimating birthweight. The results of race/ethnicity-specific cross-sectional analyses (Models 1–3, Table 4A) show protective associations between greenspace and SGA only among Black and Hispanic mothers. For example, the results of Model 3 (pooled cross-sectional analyses) show that, compared to living in a neighborhood with very low greenspace (Q1), living in a neighborhood with very high greenspace (Q4) corresponds with an 8% reduction in the odds of SGA for Black mothers (OR = 0.92, 95% CI: 0.84, 0.96) and an 11% reduction in the odds of SGA for Hispanic mothers (OR = 0.89, 95% CI: 0.87, 0.91). However, the results of within-mother analyses (Models 4–6, Table 4B) show null associations between residential greenspace and SGA across all models and race/ethnicities. Analyses examining PTB (Supplementary Table S4) and LBW (Supplementary Table S5) show substantively similar results.

## 4. Discussion

Growing epidemiologic work has examined whether greenspace in a mother’s residential environment during pregnancy reduces her risk of an adverse birth outcome, but results appear mixed [7,30,31]. Recent research has investigated explanations for inconsistent findings, including residential self-selection bias in cross-sectional work and effect modification by maternal sociodemographic characteristics [13,15]. This study aimed to understand these two potential sources of heterogeneity by using methods that minimize unmeasured confounding due to selection and assessing whether associations between residential greenspace and birth outcomes differ by maternal race/ethnicity. I find, consistent with recent work in Michigan [13], that increases in maternal exposure to residential greenspace do not predict increases in infant birthweight, controlling for unmeasured confounders, among NH white, Hispanic, and Asian mothers living in California. By contrast, results show that Black mothers who experience increases in residential greenspace between pregnancies deliver higher birthweight infants, accounting for factors that may influence both place of residence and birth outcomes. Taken together, findings suggest that residential self-selection remains a salient bias in nature-health research, while also advancing the argument that increases in residential greenspace may confer perinatal benefits for racially minoritized mothers.

Past findings on the associations between residential greenspace and perinatal health do not converge, with some studies showing more favorable birth outcomes among mothers residing in greener neighborhoods [32,33] and others showing mixed [34] or null [13] results. A unique contribution of this study includes the use of different methodological approaches to explore potential causes of inconsistent findings. In a sample of mothers who gave birth to siblings in California between 2005 and 2015, I first replicated past studies by assessing cross-sectional associations between maternal exposure to residential greenspace and birth outcomes. I then moved beyond cross-sectional methods by longitudinally tracking mothers who experienced changes in greenspace between births. The results of cross-sectional analyses show positive associations between residential greenspace and birthweight, consistent with recently reviewed research [30]. However, longitudinal maternal fixed effects analyses, which provide more robust control for unobserved confounders, indicate that increases in residential greenspace between births correspond with higher birthweight only for Black mothers. This result holds when restricting the analysis to Black mothers who do not move but experience within-neighborhood increases in greenspace over time, mimicking an in-place intervention.

Contrary to my hypothesis, results on within-mother associations between neighborhood greenspace and SGA, a birth outcome that adjusts for gestational length and gauges infant growth restriction, cannot reject the null. Findings indicate that increases in greenspace may affect birthweight but not the timing of parturition, cohering with some past studies [30]. Reduced maternal stress represents one potential pathway through which greater exposure to greenspace may improve perinatal health. A recent meta-analysis found that birthweight, more so than gestational length, responds adversely to maternal stress *in utero* [62]. The findings of this study suggest that birthweight may also respond more positively to the stress-buffering effects of greenspace.

### 4.1. Effect Modification

This study advances a small but growing body of research examining heterogeneity in relations between neighborhood greenspace and birth outcomes using maternal sociodemographic characteristics [30,38,40,41,42]. Prior work has found that the perinatal benefits of greenspace concentrate among mothers with lower education and income levels, which appears consistent with research on other health outcomes [63]. Associations between residential greenspace and cardiovascular disease mortality [64] and self-rated health [65], for example, also appear stronger (i.e., more protective) among lower socioeconomic status (SES) populations. Explanations include that, due to mobility constraints, lower SES populations spend more time in immediate neighborhood environments and, consequently, have greater exposure to, and receive more benefits from, residential greenspace [63]. Research also broadly documents worse health among lower SES groups, creating greater opportunities for health improvements following modifications to the neighborhood environment. In contrast to studies of effect modification by education level, much less work has examined whether associations between residential greenspace and birth outcomes differ by race/ethnicity. Two studies—one in Canada [41] and one in the US [40]—found no evidence of effect modification by area- or individual-level race/ethnicity. At least one study in the UK has examined racial/ethnic differences in the association between residential greenspace and birthweight, but found, in contrast to this study, a positive association among white, but not ethnic minority (e.g., Pakistani), mothers [16].

Given the substantial correlation between low SES and minoritized racial/ethnic identity in the US, socioeconomic characteristics—and not race, per se—may explain the observed protective associations between greenspace and Black births [26,38]. Descriptive statistics indicate that a higher proportion of NH Black mothers in the study sample attained less than a high school education and received public health insurance (mostly MediCAL), indicating a lower socioeconomic position, on average, among this group. In addition, consistent with the notion that lower SES populations exhibit worse health (and may have more to gain from neighborhood improvements), NH Black mothers in the US bear the greatest burden of perinatal morbidity. For example, the average birthweight among infants delivered to Black mothers in California between 2005 and 2015 was approximately 3200 g (i.e., 7 pounds [lbs] 0 ounces [oz]), compared to 3440 g (7 lbs 9 oz) among white births, as well as 3350 g (7 lbs 6 oz) among Hispanic births, and 3300 g (7 lbs 4 oz) among Asian births. However, the explanation that Black mothers differentially benefit from greenspace owing to racialized health disparities and lower socioeconomic position requires further scrutiny. A priority for future research should involve examining how race/ethnicity, socioeconomic status, and other sociodemographic characteristics (e.g., age, gender) interactively modify greenspace effects on health. This work can help identify the population subgroups who most benefit from greenspace, which can, in turn, inform how urban greening efforts should be tailored to maximize health equity impacts.

### 4.2. Strengths and Limitations

The current study diverges from past cross-sectional studies of associations between greenspace and perinatal health in several key ways. First, a principal strength of this analysis involves the use of longitudinal data and maternal fixed effects to control for time-invariant maternal characteristics that may confound greenspace–birth outcome associations. Consistent with prior work [16,30], I initially found a strong positive association between greenspace and birthweight among mothers of most racial/ethnic identities, adjusting for observed maternal and neighborhood characteristics. However, the inclusion of a mother-specific indicator variable that further controls for time-invariant maternal characteristics ameliorates this association in all but Black mothers. These findings indicate that residential selection (i.e., the process through which healthier women choose to live in greener neighborhoods) may result in spurious associations among most (i.e., non-Black) mothers in studies that cannot account for unmeasured confounders [13,14]. In addition, I restricted the fixed effects analyses to non-mobile mothers who experienced within-neighborhood changes in greenspace between births (i.e., stayers). These findings cohere with the results of unrestricted fixed effects analysis (i.e., including movers and stayers) and analysis only in movers. The consistency of results across samples minimizes the likelihood that residential selection biases this relation. Future research that emulates random assignment of mothers to conditions of greenspace [66,67] should further refine and test the hypothesized causal effect of greenspace on birth outcomes.

Another strength of this study includes the use of the California birth files for years 2005 to 2015 to provide a large and racially diverse sample of mothers. This sample was sufficient to permit theoretically motivated tests of race/ethnicity-specific differences in greenspace–birth outcome associations. Importantly, the analytic sample included over 50,000 NH Black women, who, in the US, exhibit the highest rates of adverse birth outcomes compared to all other racial/ethnic subgroups. Well-documented risk factors for adverse birth outcomes—including exposure to environmental hazards (e.g., air and noise pollution, poor water quality, and extreme heat) and heightened stress during pregnancy—disproportionately affect NH Black women [43,46]. Evidence of the unique risks and perinatal morbidities affecting NH Black women in the US, coupled with findings of the current study, support the notion that residential greenspace may contribute to increased birthweight through the provision of ecosystem services (i.e., mitigation) and stress reduction (i.e., restoration).

Limitations of this study include a lack of information on the mechanisms by which neighborhood greenspace affects birth outcomes. In addition to reducing stress and improving environmental conditions, research indicates that neighborhood greenspace may increase physical activity and social contacts (i.e., instoration) [9,11]. Given that the birth files do not contain data on these health behaviors (e.g., physical activity, social interactions), it remains unclear which (if any) of these mechanisms influenced the course of pregnancy among Black mothers in the analytic sample. In addition, this study did not include information on, or adjust for, other maternal health behaviors and conditions, such as gestational diabetes, hypertension, preeclampsia, or alcohol and tobacco use, nor did the analyses control for environmental characteristics, including air pollution or noise exposure, which may influence maternal greenspace exposure and/or infant birth outcomes. These omissions may result in residual confounding. However, environmental and maternal health conditions and behaviors may lie on the causal pathway between greenspace exposure and perinatal health, such that inclusion as covariates may bias associations towards the null. Additionally, to the extent that maternal health, behavioral, and environmental factors remain fixed across births, within-mother fixed effects analyses would control for potential unobserved confounding (though not if these factors changed over time).

Another primary limitation of this study includes the use of a home-based, census tract-level measure of residential greenspace, rather than a buffer-based approach (i.e., measuring greenspace within buffers surrounding mothers’ homes, typically ranging from 100 to 1000 m). The neighborhood-level measure used in this study may misrepresent the true spatial context through which greenspace influences perinatal health. Measuring greenspace within smaller, buffer-based units around a mother’s home may yield more accurate estimates of greenspace–birth outcome associations. Relatedly, the modifiable areal unit problem (MAUP) [68,69], which results when associations between area-level characteristics and individual-level outcomes differ based on the spatial unit of analysis, limits the comparability between the results of the present study and other research using buffer-based approaches. In addition, census tract-level measures inaccurately assume that all residents of a neighborhood have the same exposure to greenspace, regardless of their mobility levels or access limitations. While using disaggregated, home-based buffer measures may minimize this issue, such measures still overlook non-home and mobility-based exposure to greenspace.

To this end, a shift away from home-based measures and towards mobility-based measures may improve our understanding of whether and how greenspace exposure affects health in the context of everyday life [70]. For example, studies that pair mobility-based measures of greenspace exposure with ambulatory methods—including wearable biosensors, ecological momentary assessments, and accelerometers—have the potential to substantially advance knowledge of the restorative (e.g., improved mood, reduced stress) and instorative (e.g., increased physical activity) mechanisms through which greenspace affects birth outcomes. Whereas the use of mobility-based greenspace measures has grown in research on non-pregnant populations [71,72], few studies have applied such methods to track the effects of real-life greenspace exposure among pregnant people. Such work, which technological advances make increasingly accessible [73], is warranted to improve our understanding of the pathways through which greenspace exposure in home, non-home, and mobility-based settings affects perinatal health.

Finally, this study does not consider the processes through which greenspace changes within a neighborhood over time. As discussed, residential selection may threaten internal validity in studies that rely on cross-sectional data or changes in greenspace induced by moving, as residents may ‘select’ neighborhoods according to preexisting health or social factors [13,14]. By contrast, within-neighborhood changes in greenspace may serve as a plausibly exogenous exposure to the extent that vegetation levels fluctuate naturally, or changes occur randomly. However, political decisions, concerted community action, or other person-made sources of change that intervene on the environment may occur non-randomly and threaten the validity of the findings. For instance, Wolch, Byrne, and Newell [74] note that urban greening—which often involves the transformation of remnant urban land to greenspaces in low-income, under-resourced, or ‘park poor’ neighborhoods—may create paradoxical effects. Urban greening may coincide with or initiate gentrification, which increases property values and reduces housing opportunities [74]. Greening initiatives, therefore, may displace the residents they intended to benefit. Within the context of this study, the process of ‘green gentrification’ may lead to negative selection bias, as my analysis focuses on mothers who can afford to remain in transforming neighborhoods. Future work should evaluate the effects of greenspace interventions on residential mobility and displacement among low-income residents. Findings can inform the delivery of urban greening efforts to ensure that such interventions do, in fact, promote equitable access to greenspace and optimize health benefits for low-income and racially minoritized residents.

## 5. Conclusions

Inconsistent findings on associations between neighborhood greenspace and birth outcomes may derive from residential self-selection bias in cross-sectional studies or effect modification by maternal sociodemographic characteristics. This study overcomes limitations of previous work by using longitudinal data to analyze within-mother associations between changes in greenspace and birth outcomes overall and by maternal race/ethnicity. Results controlling for unmeasured maternal confounders show that infant birthweight increases among deliveries to NH Black but not NH white, Hispanic, or Asian mothers who experience increases in neighborhood greenspace. These results remain robust to analysis in a restricted sample of mothers who stay in the same neighborhood but experience within-neighborhood changes in greenspace between pregnancies. Such changes more closely mimic, from a methodological perspective, a quasi-experimental design, and, from an applied perspective, a neighborhood-level intervention. These findings suggest that greening initiatives that target historically under-resourced and racially minoritized neighborhoods may reduce persistent racial disparities in birth outcomes, an important step towards promoting health equity across the life course. The replication of results in different study populations and contexts is needed to corroborate the novel evidence presented in this study.

## Figures and Tables

**Table 1:** Characteristics of California singleton births at time 2 (sibling 2) among (A) all races/ethnicity, (B) non-Hispanic (NH) white, and (C) NH Black mothers.

	(A) All Race/Ethnicity			
	Mothers with ≥2 Births		Stayers Only ^1^	
	(*n* = 922,263)		(*n* = 489,184)	
Maternal variables	*n*	*%*	*n*	*%*
Race/ethnicity				
NH white	247,285	26.81	143,213	29.28
NH Black	54,995	5.96	21,251	4.34
NH Asian	136,414	14.79	78,952	16.14
Hispanic	463,707	50.28	235,331	48.11
Other ^2^	19,862	2.15	10,437	2.13
Age (years)				
<20	28,333	3.07	13,643	2.79
20–24	175,054	18.98	77,050	15.75
25–29	246,437	26.72	117,967	24.12
30–34	274,108	29.72	155,361	31.76
35–40	162,806	17.65	101,951	20.84
≥40	35,525	3.85	23,212	4.75
Education				
Less than high school	185,772	20.14	89,698	18.34
High school	224,241	24.31	108,120	22.1
Some college	481,281	52.18	275,075	56.23
Unknown or not stated	30,969	3.36	16,291	3.33
Insurance				
Private	460,069	49.88	271,534	55.51
Public (MediCAL)	411,178	44.58	192,095	39.27
Other ^3^	51,016	5.53	25,555	5.22
Birth variables	Median	IQR	Median	IQR
Birthweight (grams)	3380	3085–3690	3394	3090–3700
Gestational age (days)	275	269–281	275	269–281
Neighborhood variables	Median	IQR	Median	IQR
NDVI	0.53	0.43–0.62	0.53	0.43–0.62
Disadvantage	−0.05	−0.49–0.55	−0.12	−0.52–0.47
	**(B) NH White**			
	**Mothers with ≥2 Births**		**Stayers Only ^1^**	
	**(*n* = 247,285)**		**(*n* = 143,213)**	
Maternal variables	*n*	%	*n*	%
Age (years)				
<20	1998	0.81	834	0.58
20–24	24,542	9.92	9821	6.86
25–29	58,411	23.62	29,248	20.42
30–34	89,406	36.16	54,699	38.19
35–40	59,006	23.86	39,021	27.25
≥40	13,922	5.63	9590	6.7
Education				
Less than high school	9592	3.88	3880	2.71
High school	39,803	16.1	19,003	13.27
Some college	193,699	78.33	117,956	82.36
Unknown or not stated	4191	1.69	2374	1.66
Insurance				
Private	187,233	75.72	116,143	81.1
Public (MediCAL)	45,421	18.37	19,166	13.38
Other ^3^	14,631	5.92	7904	5.52
Birth variables	Median	IQR	Median	IQR
Birthweight (grams)	3480	3175–3785	3487	3187–3793
Gestational age (days)	276	271–282	276	271–282
Neighborhood variables	Median	IQR	Median	IQR
NDVI	0.55	0.48–0.64	0.56	0.48–0.64
Disadvantage	−0.40	−0.68–0.01	−0.43	−0.69–−0.06
	**(C) NH Black**			
	**Mothers with ≥2 Births**		**Stayers Only ^1^**	
	**(*n* = 54,995)**		**(*n* = 21,251)**	
Maternal variables	*n*	*%*	*n*	*%*
Age (years)				
<20	2399	4.36	920	4.33
20–24	15,708	28.56	5251	24.71
25–29	16,705	30.38	5849	27.52
30–34	12,397	22.54	5216	24.54
35–40	6251	11.37	3157	14.86
≥40	1535	2.79	858	4.04
Education				
Less than high school	8087	14.7	2653	12.48
High school	18,044	32.81	6449	30.35
Some college	27,627	50.24	11,701	55.06
Unknown or not stated	1237	2.25	448	2.11
Insurance				
Private	18,784	34.16	8745	41.15
Public (MediCAL)	31,465	57.21	10,599	49.88
Other ^3^	4746	8.63	1907	8.97
Birth variables	Median	IQR	Median	IQR
Birthweight (grams)	3250	2929–3572	3275	2948–3600
Gestational age (days)	274	267–281	274	268–281
Neighborhood variables	Median	IQR	Median	IQR
NDVI	0.51	0.35–0.62	0.49	0.35–0.62
Disadvantage	0.47	−0.10–1.13	0.36	−0.19–1.01

Note. ^1^ The ‘stayers only’ sample includes mothers with at least two live births who did not move between births. ^2^ Other maternal race/ethnicity includes American Indian or Alaskan Native, Native Hawaiian or other Pacific Islander, more than one race reported, and unknown or not stated. ^3^ Other insurance provider includes self-pay, Indian Health Service, other governmental (federal, state, local), and unknown or not stated. Abbreviations: IQR, interquartile range; NDVI, Normalized Difference Vegetation Index; NH, non-Hispanic.

**Table 2:** Distribution of mothers according to quartiles of neighborhood greenspace (NDVI) at time 1 (sibling 1) and time 2 (sibling 2) among (A) all race/ethnicity, (B) non-Hispanic (NH) white, and (C) NH Black mothers.

	(A) All Race/Ethnicity			
	Time 1		Time 2	
	*n*	%	*n*	%
Q1 (very low)	244,562	26.52	240,209	26.05
Q2	233,163	25.28	230,866	25.03
Q3	230,618	25.01	232,158	25.17
Q4 (very high)	213,920	23.20	219,027	23.75
	**(B)** **NH White**			
	**Time 1**		**Time 2**	
	*n*	%	*n*	%
Q1 (very low)	41,222	16.68	41,754	16.89
Q2	63,307	25.62	60,617	24.51
Q3	74,124	30.00	75,821	30.66
Q4 (very high)	68,419	27.69	69,092	27.94
	**(C)** **NH Black**			
	**Time 1**		**Time 2**	
	*n*	%	*n*	%
Q1 (very low)	21,365	38.33	21,064	38.30
Q2	10,349	18.57	9560	17.38
Q3	11,215	20.12	11,175	20.32
Q4 (very high)	12,804	22.97	13,196	23.99

Abbreviations: NDVI, Normalized Difference Vegetation Index; NH, non-Hispanic; Q, quartile.

**Table 3:** Coefficients (coef.) and 95% confidence intervals (CI) predicting infant birthweight (in grams) as a function of residential greenspace (NDVI), overall and by race/ethnicity, among mothers with at least two live births in California, 2005–2015.

	(A) Cross-Sectional Analyses								
	Model 1			Model 2			Model 3		
	Coef.	CI		Coef.	CI		Coef.	CI	
Race/ethnicity									
All	97.39	89.37,	105.40	68.64	60.32,	76.96	73.47	67.07,	79.86
NH white	59.09	42.81,	75.38	52.62	35.87,	69.37	68.08	55.61,	81.56
NH Black	120.33	88.63,	152.03	97.18	64.99,	129.38	92.60	67.68,	117.51
Hispanic	110.18	98.79,	121.57	93.78	82.01,	105.55	93.72	84.63,	102.81
Asian	9.13	−10.66,	28.93	5.49	−14.87,	25.86	12.54	−3.18,	28.26
Sample includes:									
Sibling 1 (time 1)	Yes			Yes			Yes		
Sibling 2 (time 2)	No			No			Yes		
Stayers	N/A			N/A			N/A		
Movers	N/A			N/A			N/A		
Adjusted for:									
Year	No			Yes			Yes		
Maternal factors	No			Yes			Yes		
Neighborhood factors	No			Yes			Yes		
Maternal fixed effects:	No			No			No		
	**(B) Within-Mother Analyses**								
	**Model 4**			**Model 5**			**Model 6**		
	**Coef.**	**CI**		**Coef.**	**CI**		**Coef.**	**CI**	
Race/ethnicity									
All	8.19	−3.41,	19.81	−1.63	−16.74,	20.01	12.80	−2.30,	27.90
NH white	−0.51	−22.94,	21.91	−18.60	−52.25,	15.04	14.35	−16.04,	44.74
NH Black	75.49	23.48,	127.50	114.07	11.58,	216.58	62.09	1.50,	122.68
Hispanic	13.32	−3.95,	30.58	14.67	−13.38,	42.72	12.79	−9.15,	34.73
Asian	−9.72	−37.91,	18.47	2.89	−40.37,	46.15	−16.45	−53.76,	20.86
Sample includes:									
Sibling 1 (time 1)	Yes			Yes			Yes		
Sibling 2 (time 2)	Yes			Yes			Yes		
Stayers	Yes			Yes			No		
Movers	Yes			No			Yes		
Adjusted for:									
Year	Yes			Yes			Yes		
Maternal factors	Yes			Yes			Yes		
Neighborhood factors	Yes			Yes			Yes		
Maternal fixed effects:	Yes			Yes			Yes		

Abbreviations: CI, confidence interval; coef., coefficient; NH, non-Hispanic.

**Table 4:** Odds ratio (OR) and 95% confidence intervals (CI) predicting the probability of a small-for-gestational age (SGA) birth as a function of residential greenspace (NDVI, in quartiles), overall and by race/ethnicity, among mothers with at least two live births in California, 2005–2015.

		(A) Cross-Sectional Analyses								
		Model 1			Model 2			Model 3		
		OR	CI		OR	CI		OR	CI	
Race/ethnicity										
All	Q2 (vs. Q1)	0.93	0.92,	0.95	0.96	0.94,	0.97	0.97	0.95,	0.98
	Q3 (vs. Q1)	0.91	0.88,	0.93	0.94	0.92,	0.95	0.95	0.94,	0.97
	Q4 (vs. Q1)	0.92	0.92,	0.94	0.96	0.96,	0.98	0.93	0.92,	0.94
NH white	Q2 (vs. Q1)	0.95	0.91,	1.00	0.96	0.92,	1.00	0.95	0.92,	1.00
	Q3 (vs. Q1)	0.96	0.92,	1.00	0.97	0.93,	1.01	0.95	0.92,	1.00
	Q4 (vs. Q1)	0.96	0.92,	1.00	0.97	0.93,	1.01	0.95	0.92,	1.00
NH Black	Q2 (vs. Q1)	1.00	0.94,	1.06	1.03	0.97,	1.09	1.01	0.97,	1.05
	Q3 (vs. Q1)	0.91	0.86,	0.97	0.93	0.88,	0.99	0.96	0.88,	0.99
	Q4 (vs. Q1)	0.86	0.81,	0.91	0.89	0.84,	0.95	0.92	0.84,	0.96
Hispanic	Q2 (vs. Q1)	0.95	0.92,	0.97	0.96	0.94,	0.98	0.96	0.95,	0.98
	Q3 (vs. Q1)	0.93	0.90,	0.95	0.94	0.92,	0.97	0.95	0.93,	0.96
	Q4 (vs. Q1)	0.88	0.86,	0.90	0.90	0.88,	0.92	0.89	0.87,	0.91
Asian	Q2 (vs. Q1)	1.02	0.98,	1.07	1.03	0.99,	1.07	1.01	0.97,	1.04
	Q3 (vs. Q1)	1.01	0.97,	1.05	1.01	0.97,	1.06	0.99	0.96,	1.02
	Q4 (vs. Q1)	1.03	0.98,	1.06	1.03	0.99,	1.07	1.00	0.97,	1.04
Sample includes:										
Sibling 1 (time 1)		Yes			Yes			Yes		
Sibling 2 (time 2)		No			No			Yes		
Stayers		N/A			N/A			N/A		
Movers		N/A			N/A			N/A		
Adjusted for:										
Year		No			Yes			Yes		
Maternal factors		No			Yes			Yes		
Neighborhood factors		No			Yes			Yes		
Maternal fixed effects:		No			No			No		
		**(B) Within-Mother Analyses**								
		**Model 4**			**Model 5**			**Model 6**		
		**OR**	**CI**		**OR**	**CI**		**OR**	**CI**	
Race/ethnicity										
All	Q2 (vs. Q1)	0.97	0.94,	1.00	0.99	0.95,	1.03	0.96	0.92,	1.00
	Q3 (vs. Q1)	1.00	0.97,	1.03	1.00	0.95,	1.06	0.99	0.95,	1.04
	Q4 (vs. Q1)	0.99	0.95,	1.03	0.98	0.92,	1.05	0.99	0.94,	1.04
NH white	Q2 (vs. Q1)	0.94	0.88,	1.01	0.92	0.83,	1.03	0.97	0.87,	1.07
	Q3 (vs. Q1)	0.98	0.90,	1.06	0.99	0.88,	1.12	0.97	0.87,	1.08
	Q4 (vs. Q1)	0.99	0.90,	1.08	1.00	0.88,	1.15	0.97	0.86,	1.09
NH Black	Q2 (vs. Q1)	1.10	0.98,	1.22	1.03	0.84,	1.27	1.11	0.98,	1.27
	Q3 (vs. Q1)	0.97	0.86,	1.09	0.86	0.67,	1.09	1.01	0.88,	1.16
	Q4 (vs. Q1)	0.96	0.83,	1.10	0.93	0.71,	1.24	0.97	0.82,	1.13
Hispanic	Q2 (vs. Q1)	0.97	0.93,	1.01	0.99	0.93,	1.05	0.95	0.90,	1.00
	Q3 (vs. Q1)	1.02	0.98,	1.07	1.00	0.93,	1.08	1.04	0.98,	1.10
	Q4 (vs. Q1)	1.02	0.96,	1.08	0.96	0.87,	1.05	1.05	0.98,	1.13
Asian	Q2 (vs. Q1)	1.00	0.93,	1.07	1.05	0.96,	1.16	0.95	0.86,	1.05
	Q3 (vs. Q1)	1.01	0.93,	1.09	1.05	0.94,	1.17	0.97	0.87,	1.08
	Q4 (vs. Q1)	0.98	0.90,	1.07	1.00	0.87,	1.14	0.96	0.85,	1.08
Sample includes:										
Sibling 1 (time 1)		Yes			Yes			Yes		
Sibling 2 (time 2)		Yes			Yes			Yes		
Stayers		Yes			Yes			No		
Movers		Yes			No			Yes		
Adjusted for:										
Year		Yes			Yes			Yes		
Maternal factors		Yes			Yes			Yes		
Neighborhood factors		Yes			Yes			Yes		
Maternal fixed effects:		Yes			Yes			Yes		

Abbreviations: CI, confidence interval; Coef., coefficient; NDVI, Normalized Difference Vegetation Index; NH, non-Hispanic.

## Data Availability

The data underlying this article cannot be shared publicly to guarantee the anonymity of subjects. Code is available upon request.

## Supplementary Materials

The following supporting information can be downloaded at: https://www.mdpi.com/article/10.3390/ijerph20186790/s1, Table S1. Mean change (STD) in neighborhood greenspace between births among all mothers with two or more live births and stayers only by maternal race/ethnicity; Table S2. Coefficients (coef.) and 95% confidence intervals (CI) predicting infant birthweight (in grams) as a function of a 0.10-unit increase in residential greenspace (NDVI), overall and by race/ethnicity, among mothers with at least two live births in California, 2005–2015; Table S3. Coefficients (coef.) and 95% confidence intervals predicting infant birthweight (in grams) as a function of an interquartile range (IQR) increase (IQR = 0.194) in residential greenspace (NDVI), overall and by race/ethnicity, among mothers with at least two live births in California, 2005–2015; Table S4. Odds ratios (OR) and 95% confidence intervals (CI) predicting the probability of a preterm birth (PTB) as a function of residential greenspace (NDVI, in quartiles), overall and by race/ethnicity, among mothers with at least two live births in California, 2005–2015; Table S5. Odds ratios (OR) and 95% confidence intervals (CI) predicting the probability of a low birthweight (LBW) birth as a function of residential greenspace (NDVI, in quartiles), overall and by race/ethnicity, among mothers with at least two live births in California, 2005–2015. References [75,76,77,78] are cited in the Supplementary Materials.
