# Supplementary material for: Changes in Residential Greenspace and Birth Outcomes among Siblings: Differences by Maternal Race
Source: Int J Environ Res Public Health. 2023 Sep 21;20(18):6790. doi: 10.3390/ijerph20186790 (PMC10531468; doi:10.3390/ijerph20186790)
Supplement: Supplementary file 1 [file ijerph-20-06790-s001.zip › ijerph-2506487-supplementary.pdf]

Supplementary Material

Link Plus Methods

Link Plus implements a probabilistic record linkage algorithm developed by Fellegi and Sunter [1], called the Fellegi-Sunter methodology; the formal mathematical models involved in this methodology underpin most modern record linkages (see Herzog and Scheuren [2] for review). A recent study [3] comparing linkage methods commonly used in public health reports that programs based on the Fellegi-Sunter method (including, but not limited to, Link Plus) maximize the number of true matches identified, given their high degree of sensitivity (i.e., the proportion of true matches identified by the algorithm) and precision (i.e., the proportion of matches identified by the algorithm that were true matches). By contrast, programs based on deterministic matching algorithms exhibit high precision but low sensitivity, and thus perform poorly when using lower quality data (i.e., misspelled or missing fields). Such programs may induce systematic bias due to the exclusion of record-linkages for lower SES and racial-ethnic minority populations [4].

In general, Link Plus proceeds through the following steps. First, the program identifies potential matches by 'blocking' record pairs with exact values on a user-specified field. These comparison-pairs then receive a match score based on similarity of specified 'match' variables; pairs with higher scores appear more likely to reflect 'true' matches. Next, the user sets a lower-bound match score, above which he / she / they can review pairs and assign designations of 'true match,' 'uncertain,' or 'no match.' Pairs with match scores below the specified lower-bound receive a 'no match' designation and are dropped.

I used Link Plus to 'block' records based on birthing persons' date of birth. Potential matches (i.e., all record pairs with the same date of birth) then received match scores according to similarity of a birthing person's first and last name, as well as the other parent's date of birth. Match scores ranged from 0 (pairs with the same birthing person's date of birth but differing on all other variables) to approximately 25 (pairs matching on all variables). In order to consider a broad range of matches and avoid dropping potential low-scoring 'true matches,' I set a lower-bound match score of 5.0, above which comparison-pairs received a temporary designation of 'uncertain.'

I created user-defined categories to sort these remaining 'uncertain' comparison-pairs. I assigned pairs matching on all variables to one category, pairs matching on birthing person's last name and other parent's date of birth (but not birthing person's first name) to a second category, and those matching on birthing person's first and last name (but not other parent's date of birth) to a third category. I excluded from review comparison-pairs not included in these

three categories. I designated pairs in the first category (matching on all variables) as ‘true matches.’ For pairs in the second and third category, I assigned a ‘true match’ designation only to those in which the date of birth for record 1 (first sibling) corresponded with the date of last delivery for record 2 (second sibling). This process yielded 1,340,676 ‘true match’ pairs, representing birthing persons with at least two consecutive live singleton births in California between 2005 and 2015.

References

1. Fellegi, I.P.; Sunter, A.B. A Theory for Record Linkage. *J. Am. Stat. Assoc.* **1969**, *64*, 1183–1210, doi:10.1080/01621459.1969.10501049.
2. Herzog, T.N.; Scheuren, F.J.; Winkler, W.E. *Data Quality and Record Linkage Techniques*; Springer Science & Business Media, 2007; ISBN 978-0-387-69505-1.
3. Avoundjian, T.; Dombrowski, J.C.; Golden, M.R.; Hughes, J.P.; Guthrie, B.L.; Baseman, J.; Sadinle, M. Comparing Methods for Record Linkage for Public Health Action: Matching Algorithm Validation Study. *JMIR Public Health Surveill.* **2020**, *6*, e15917, doi:10.2196/15917.
4. Bohensky, M.A.; Jolley, D.; Sundararajan, V.; Evans, S.; Pilcher, D.V.; Scott, I.; Brand, C.A. Data Linkage: A Powerful Research Tool with Potential Problems. *BMC Health Serv. Res.* **2010**, *10*, 346, doi:10.1186/1472-6963-10-346.

Supplementary Table S1. Mean change (STD) in neighborhood greenspace between births among all mothers with two or more live births and stayers only by maternal race/ethnicity.

	Mothers with ≥ 2 births		Stayers only	
	Mean	STD	Mean	STD
All race/ethnicity	0.01	(0.10)	0.01	(0.09)
NH white	-0.01	(0.10)	-0.01	(0.10)
NH Black	0.03	(0.11)	0.02	(0.09)

Abbreviations: NH, non-Hispanic; STD, standard deviation.

Supplementary Table S2. Coefficients (coef.) and 95% confidence intervals (CI) predicting infant birthweight (in grams) as a function of a 0.10-unit increase in residential greenspace (NDVI), overall and by race/ethnicity, among mothers with at least two live births in California, 2005-2015.

(A) Cross-sectional analyses

	**Model 1**		**Model 2**		**Model 3**	
	Coef.	CI	Coef.	CI	Coef.	CI
Race/ethnicity						
All	9.74	8.94, 10.54	6.86	6.03, 7.70	7.35	6.71, 7.99
NH white	5.91	4.28, 7.54	5.26	3.59, 6.94	6.81	5.56, 8.16
NH Black	12.03	8.86, 15.20	9.72	6.50, 12.94	9.26	6.77, 11.75
Hispanic	11.02	9.88, 12.16	9.38	8.20, 10.56	9.37	8.46, 10.28
Asian	0.91	-1.07, 2.89	0.55	-1.49, 2.59	1.25	-0.32, 2.83
Sample includes:						
Sibling 1 (time 1)	Yes		Yes		Yes	
Sibling 2 (time 2)	No		No		Yes	
Stayers	N/A		N/A		N/A	
Movers	N/A		N/A		N/A	
Adjusted for:						
Year	No		Yes		Yes	
Maternal factors	No		Yes		Yes	
Neighborhood factors	No		Yes		Yes	
Maternal fixed effects:	No		No		No	

(B) Within-mother analyses

	Model 4		Model 5		Model 6	
	Coef.	CI	Coef.	CI	Coef.	CI
Race/ethnicity						
All	0.82	-0.34, 1.98	-0.16	-1.67, 2.00	1.28	-0.23, 2.79
NH white	-0.05	-2.29, 2.19	-1.86	-5.23, 1.50	1.44	-1.60, 4.47
NH Black	7.55	2.35, 12.75	11.41	1.16, 21.66	6.21	0.15, 12.27
Hispanic	1.33	-0.40, 3.06	1.47	-1.34, 4.27	1.28	-0.92, 3.47
Asian	-0.97	-3.79, 1.85	0.29	-4.04, 4.62	-1.65	-5.38, 2.09
Sample includes:						
Sibling 1 (time 1)	Yes		Yes		Yes	
Sibling 2 (time 2)	Yes		Yes		Yes	
Stayers	Yes		Yes		No	
Movers	Yes		No		Yes	
Adjusted for:						
Year	Yes		Yes		Yes	
Maternal factors	Yes		Yes		Yes	
Neighborhood factors	Yes		Yes		Yes	
Maternal fixed effects:	Yes		Yes		Yes	

Abbreviations: CI, confidence interval; Coef., coefficient; NH, non-Hispanic.

Supplementary Table S3. Coefficients (coef.) and 95% confidence intervals predicting infant birthweight (in grams) as a function of an interquartile range (IQR) increase (IQR=0.194) in residential greenspace (NDVI), overall and by race/ethnicity, among mothers with at least two live births in California, 2005-2015.

(A) Cross-sectional analyses

	**Model 1**			**Model 2**			**Model 3**		
	Coef.	CI		Coef.	CI		Coef.	CI	
Race/ethnicity									
All	18.90	17.34,	20.45	13.32	11.71,	14.94	14.26	13.02,	15.50
NH white	11.47	8.31,	14.63	10.21	6.96,	13.46	13.21	10.79,	15.83
NH Black	23.35	17.20,	29.50	18.86	12.61,	25.11	17.97	13.13,	22.81
Hispanic	21.38	19.17,	23.59	18.20	15.92,	20.48	18.19	16.42,	19.95
Asian	1.77	-2.07,	5.61	1.07	-2.89,	5.02	2.43	-0.62,	5.48
Sample includes:									
Sibling 1 (time 1)	Yes			Yes			Yes		
Sibling 2 (time 2)	No			No			Yes		
Stayers	N/A			N/A			N/A		
Movers	N/A			N/A			N/A		
Adjusted for:									
Year	No			Yes			Yes		
Maternal factors	No			Yes			Yes		
Neighborhood factors	No			Yes			Yes		
Maternal fixed effects:	No			No			No		

(B) Within-mother analyses

	Model 4		Model 5		Model 6	
	Coef.	CI	Coef.	CI	Coef.	CI
Race/ethnicity						
All	1.59	-0.66, 3.84	-0.32	-3.25, 3.88	2.48	-0.45, 5.41
NH white	-0.10	-4.45, 4.25	-3.61	-10.14, 2.92	2.78	-3.11, 8.68
NH Black	14.65	4.56, 24.74	22.14	2.25, 42.03	12.05	0.29, 23.81
Hispanic	2.59	-0.77, 5.93	2.85	-2.60, 8.29	2.48	-1.78, 6.74
Asian	-1.89	-7.36, 3.58	0.56	-7.83, 8.96	-3.19	-10.43, 4.05
Sample includes:						
Sibling 1 (time 1)	Yes		Yes		Yes	
Sibling 2 (time 2)	Yes		Yes		Yes	
Stayers	Yes		Yes		No	
Movers	Yes		No		Yes	
Adjusted for:						
Year	Yes		Yes		Yes	
Maternal factors	Yes		Yes		Yes	
Neighborhood factors	Yes		Yes		Yes	
Maternal fixed effects:	Yes		Yes		Yes	

Abbreviations: CI, confidence interval; Coef., coefficient; NH, non-Hispanic.

Supplementary Table S4. Odds ratios (OR) and 95% confidence intervals (CI) predicting the probability of a preterm birth (PTB) as a function of residential greenspace (NDVI, in quartiles), overall and by race/ethnicity, among mothers with at least two live births in California, 2005-2015.

(A) Cross-sectional analyses

		**Model 1**		**Model 2**		**Model 3**	
		OR	CI	OR	CI	OR	CI
Race/ethnicity							
	Q2 (vs. Q1)	0.90	0.88, 0.92	0.95	0.93, 0.97	0.97	0.96, 0.99
All	Q3 (vs. Q1)	0.92	0.90, 0.94	0.97	0.95, 0.99	0.99	0.98, 1.01
	Q4 (vs. Q1)	0.85	0.83, 0.86	0.92	0.90, 0.94	0.92	0.91, 0.94
	Q2 (vs. Q1)	1.02	0.97, 1.08	1.03	0.98, 1.08	1.03	0.98, 1.06
NH white	Q3 (vs. Q1)	1.03	0.98, 1.08	1.02	0.97, 1.09	1.02	0.98, 1.05
	Q4 (vs. Q1)	1.02	0.97, 1.07	1.02	0.97, 1.07	1.02	0.94, 1.01
	Q2 (vs. Q1)	0.94	0.87, 1.01	0.95	0.88, 1.03	0.93	0.88, 0.98
NH Black	Q3 (vs. Q1)	0.98	0.91, 1.06	0.99	0.92, 1.07	1.00	0.94, 1.05
	Q4 (vs. Q1)	0.86	0.80, 0.92	0.88	0.82, 0.95	0.89	0.84, 0.94
	Q2 (vs. Q1)	0.92	0.89, 0.94	0.95	0.92, 0.97	0.95	0.93, 0.97
Hispanic	Q3 (vs. Q1)	0.97	0.93, 0.99	0.99	0.96, 1.02	0.99	0.97, 1.01
	Q4 (vs. Q1)	0.87	0.84, 0.90	0.90	0.87, 0.93	0.91	0.89, 0.93
	Q2 (vs. Q1)	1.00	0.94, 1.07	1.00	0.94, 1.07	1.01	0.97, 1.06
Asian	Q3 (vs. Q1)	1.01	0.95, 1.07	0.99	0.93, 1.05	0.97	0.92, 1.01
	Q4 (vs. Q1)	0.93	0.93, 0.99	0.94	0.89, 1.00	0.92	0.88, 0.97
Sample includes:							
Sibling 1 (time 1)		Yes		Yes		Yes	
Sibling 2 (time 2)		No		No		Yes	
Stayers		N/A		N/A		N/A	
Movers		N/A		N/A		N/A	
Adjusted for:							
Year		No		Yes		Yes	
Maternal factors		No		Yes		Yes	
Neighborhood factors		No		Yes		Yes	
Maternal fixed effects:		No		No		No	

(B) Within-mother analyses

		Model 4			Model 5			Model 6		
		OR	CI		OR	CI		OR	CI	
Race/ethnicity										
	Q2 (vs. Q1)	0.99	0.96,	1.02	1.00	0.95,	1.05	0.99	0.95,	1.03
All	Q3 (vs. Q1)	1.03	0.99,	1.07	1.04	0.98,	1.10	1.03	0.98,	1.08
	Q4 (vs. Q1)	0.99	0.94,	1.03	0.99	0.93,	1.06	0.98	0.93,	1.04
	Q2 (vs. Q1)	0.97	0.89,	1.05	1.00	0.89,	1.12	0.94	0.84,	1.06
NH white	Q3 (vs. Q1)	0.98	0.90,	1.07	1.05	0.92,	1.19	0.92	0.82,	1.03
	Q4 (vs. Q1)	0.93	0.84,	1.03	0.96	0.83,	1.12	1.00	0.79,	1.03
	Q2 (vs. Q1)	0.95	0.83,	1.08	0.97	0.76,	1.23	0.93	0.80,	1.09
NH Black	Q3 (vs. Q1)	1.01	0.88,	1.16	0.87	0.66,	1.15	1.06	0.90,	1.25
	Q4 (vs. Q1)	0.99	0.99,	1.17	0.84	0.61,	1.15	1.05	0.87,	1.26
	Q2 (vs. Q1)	1.00	0.96,	1.04	0.99	0.93,	1.05	1.00	0.95,	1.06
Hispanic	Q3 (vs. Q1)	1.07	1.02,	1.12	1.04	0.96,	1.13	1.09	1.02,	1.16
	Q4 (vs. Q1)	1.00	0.94,	1.07	1.01	0.91,	1.11	1.00	0.92,	1.08
	Q2 (vs. Q1)	1.00	0.92,	1.09	1.01	0.89,	1.14	0.99	0.88,	1.13
Asian	Q3 (vs. Q1)	0.98	0.88,	1.08	0.99	0.86,	1.15	0.95	0.83,	1.09
	Q4 (vs. Q1)	0.94	0.84,	1.05	0.96	0.81,	1.14	0.93	0.80,	1.08
Sample includes:										
Sibling 1 (time 1)		Yes			Yes			Yes		
Sibling 2 (time 2)		Yes			Yes			Yes		
Stayers		Yes			Yes			No		
Movers		Yes			No			Yes		
Adjusted for:										
Year		Yes			Yes			Yes		
Maternal factors		Yes			Yes			Yes		
Neighborhood factors		Yes			Yes			Yes		
Maternal fixed effects:		Yes			Yes			Yes		

Abbreviations: CI, confidence interval; Coef., coefficient; NDVI, Normalized Difference Vegetation Index; NH, non-Hispanic.

Supplementary Table S5. Odds ratios (OR) and 95% confidence intervals (CI) predicting the probability of a low birthweight (LBW) birth as a function of residential greenspace (NDVI, in quartiles), overall and by race/ethnicity, among mothers with at least two live births in California, 2005-2015.

(A) Cross-sectional analyses

		Model 1		Model 2		Model 3	
		OR	CI	OR	CI	OR	CI
Race/ethnicity							
All	Q2 (vs. Q1)	0.91	0.89, 0.93	0.94	0.92, 0.97	0.96	0.94, 0.98
	Q3 (vs. Q1)	0.91	0.91, 0.93	0.94	0.92, 0.97	0.98	0.96, 0.96
	Q4 (vs. Q1)	0.92	0.92, 0.94	0.97	0.94, 0.99	0.95	0.93, 0.97
NH white	Q2 (vs. Q1)	0.96	0.90, 1.02	0.97	0.91, 1.04	0.95	0.90, 0.99
	Q3 (vs. Q1)	0.96	0.90, 1.02	0.97	0.91, 1.03	0.97	0.95, 1.01
	Q4 (vs. Q1)	0.97	0.91, 1.04	0.98	0.92, 1.05	0.95	0.90, 0.99
NH Black	Q2 (vs. Q1)	0.99	0.91, 1.08	1.02	0.94, 1.12	0.97	0.91, 1.03
	Q3 (vs. Q1)	0.96	0.89, 1.05	0.99	0.91, 1.08	1.00	0.95, 1.01
	Q4 (vs. Q1)	0.92	0.85, 0.99	0.95	0.87, 1.03	0.94	0.88, 0.99
Hispanic	Q2 (vs. Q1)	0.94	0.90, 0.97	0.95	0.92, 0.99	0.96	0.93, 0.98
	Q3 (vs. Q1)	0.94	0.90, 0.97	0.95	0.92, 0.99	0.97	0.94, 1.00
	Q4 (vs. Q1)	0.91	0.87, 0.94	0.93	0.89, 0.96	0.92	0.89, 0.95
Asian	Q2 (vs. Q1)	1.01	0.94, 1.08	1.02	0.95, 1.09	1.01	0.97, 1.06
	Q3 (vs. Q1)	1.03	0.96, 1.12	1.03	0.96, 1.11	0.97	0.92, 1.01
	Q4 (vs. Q1)	1.05	0.89, 1.12	1.05	0.99, 1.13	0.92	0.88, 0.97
Sample includes:							
Sibling 1 (time 1)		Yes		Yes		Yes	
Sibling 2 (time 2)		No		No		Yes	
Stayers		N/A		N/A		N/A	
Movers		N/A		N/A		N/A	
Adjusted for:							
Year		No		Yes		Yes	
Maternal factors		No		Yes		Yes	
Neighborhood factors		No		Yes		Yes	
Maternal fixed effects:		No		No		No	

(B) Within-mother analyses

		Model 4			Model 5			Model 6		
		OR	CI		OR	CI		OR	CI	
Race/ethnicity										
	Q2 (vs. Q1)	0.96	0.96,	0.91	0.98	0.92,	1.05	0.94	0.88,	0.99
All	Q3 (vs. Q1)	0.98	1.00,	1.05	1.01	0.94,	1.10	0.98	0.92,	1.04
	Q4 (vs. Q1)	0.95	0.99,	1.05	0.98	0.89,	1.08	0.99	0.92,	1.06
	Q2 (vs. Q1)	0.91	0.82,	1.02	0.98	0.83,	1.15	0.86	0.74,	1.00
NH white	Q3 (vs. Q1)	0.94	0.83,	1.06	1.05	0.98,	1.25	0.86	0.73,	1.01
	Q4 (vs. Q1)	0.90	0.79,	1.03	1.01	0.82,	1.23	0.82	0.69,	0.99
	Q2 (vs. Q1)	0.87	0.74,	1.02	0.82	0.61,	1.11	0.88	0.74,	1.07
NH Black	Q3 (vs. Q1)	0.94	0.80,	1.12	0.84	0.60,	1.18	0.97	0.80,	1.18
	Q4 (vs. Q1)	0.92	0.76,	1.12	0.89	0.60,	1.32	0.93	0.74,	1.16
	Q2 (vs. Q1)	0.92	0.87,	0.98	0.93	0.85,	1.02	0.91	0.84,	0.98
Hispanic	Q3 (vs. Q1)	1.00	0.94,	1.07	0.95	0.85,	1.07	1.03	0.94,	1.12
	Q4 (vs. Q1)	0.98	0.90,	1.07	0.87	0.76,	1.00	1.06	0.95,	1.18
	Q2 (vs. Q1)	1.12	1.00,	1.24	1.09	0.94,	1.27	1.16	1.00,	1.35
Asian	Q3 (vs. Q1)	1.08	0.95,	1.21	1.09	0.91,	1.29	1.06	0.90,	1.26
	Q4 (vs. Q1)	1.13	0.99,	1.30	1.14	0.93,	1.40	1.13	0.94,	1.35
Sample includes:										
Sibling 1 (time 1)		Yes			Yes			Yes		
Sibling 2 (time 2)		Yes			Yes			Yes		
Stayers		Yes			Yes			No		
Movers		Yes			No			Yes		
Adjusted for:										
Year		Yes			Yes			Yes		
Maternal factors		Yes			Yes			Yes		
Neighborhood factors		Yes			Yes			Yes		
Maternal fixed effects:		Yes			Yes			Yes		

Abbreviations: CI, confidence interval; Coef., coefficient; NDVI, Normalized Difference Vegetation Index; NH, non-Hispanic.
